# Cancer Rate of the Indeterminate Lesions at Low or High Risk According to Italian System for Reporting of Thyroid FNA

**DOI:** 10.3389/fendo.2018.00371

**Published:** 2018-07-10

**Authors:** Stefano Valabrega, Giuliano Santolamazza, Francesco Romanelli, Giorgia Scapicchio, Francesco D'Angelo, Carlo Bellotti, Paolo Aurello, Luciano Izzo, Maria R. Giovagnoli, Pierpaolo Trimboli

**Affiliations:** ^1^Department of Medical and Surgical Sciences, Ospedale S. Andrea, Sapienza University, Rome, Italy; ^2^Department of Experimental Medicine, Sapienza University, Rome, Italy; ^3^Department of Surgery “P. Valdoni, Sapienza University, Rome, Italy; ^4^Department of Clinical and Molecular Medicine, Ospedale S. Andrea, Sapienza University, Rome, Italy; ^5^Department of Nuclear Medicine and Thyroid Centre, Oncology Institute of Southern Switzerland, Bellinzona, Switzerland

**Keywords:** fine needle aspiration (FNA), indeterminate, thyroid, carcinoma, nodule

## Abstract

**Background:** Italian consensus for the classification and reporting of thyroid cytology (ICCRTC) has been used in almost all Italian institutions since 2014. High reliability of ICCRTC in classifying low and high risk indeterminate nodules (Tir 3A and Tir 3B, respectively) was demonstrated. Here we reviewed our casuistry of thyroid indeterminate lesions to analyze the histologic outcome.

**Methods:** All lesions undergone FNA and final histology at S. Andrea Hospital of Rome after a cytologic assessment of Tir 3A and Tir 3B, according to ICCRTC, were included in the study.

**Results:** A number of 157 indeterminate FNA was found after the introduction of ICCRTC. Of these, 75 undergone surgery and were finally included for the study. At histology we found a 33.3% of cancers and a 67.7% of benign lesions. Out of the overall series, 25 were classified as Tir 3A and 50 as Tir 3B. Cancer rate observed in Tir 3A (1/25, 4%) was significantly (*p* = 0.0002) lower than that of Tir 3B (24/50, 48%). No significant difference was found in age and size between the two subcategories.

**Conclusions:** We confirm in our series that Italian consensus for the classification and reporting of thyroid cytology allows to discriminate indeterminate lesions at low and high risk of malignancy.

## Introduction

Cytology from fine-needle aspiration (FNA) is recognized as the pivotal evaluation of thyroid nodules to detect malignancy and benign lesions (1, 2). The most significant limit of FNA is represented by indeterminate cytologic report which occurs in up to 20–25% of all FNA. Out of these cases, only a rate of one in four or one in three is expected to be a cancer at final histology. Thus, to preoperatively diagnose thyroid nodules cytologically classified as indeterminate has represented one major challenge (3). Even if many imaging or molecular parameters have been investigated (4–14), none has reached the final evidence to be used in clinical practice.

During the last years the most important international societies have revised their guidelines for reporting thyroid cytology in the attempt to discriminate indeterminate lesions at high risk of malignancy and that at low risk, in which surgery or clinical follow-up should be considered, respectively (15, 16). More recently, the Italian consensus for the classification and reporting of thyroid cytology (ICCRTC) reported a five-classes system with a subclassification of Tir 3 in Tir 3A (low risk) and Tir 3B (high risk) (17). In the Italian system, Tir 3A is defined as increased cellularity with numerous microfollicular structures in a background of scant colloid; this category also includes partially compromised samples such as preparation artifacts or blood contamination; on the other hand Tir 3B is featured by high cellularity in a monotonous microfollicular/trabecular arrangement, with scant or absent colloid, suggestive for follicular neoplasm (3); furthermore, ICCRTC includes in the subcategory Tir 3B those cases with “mild/focal nuclear atypia,” while British and Bethesda classifications include the atypia in their categories of indeterminate at low risk [i.e., Thy 3a (15) and Category III (16), respectively]. Importantly, relevant findings were found in a meta-analysis on the reliability of ICCRTC to select high and low risk lesions, being the rate of malignancy of Tir 3A significantly lower than that of Tir 3B (18).

At our institute the most updated version of Italian system for reporting thyroid cytology (17) has been used since its publication, and the patients have been managed according to these reports. The above mentioned interesting results of ICCRTC (18) prompted us to review our series of indeterminate nodules and verify these data.

## Materials and methods

### Selection of patients

We initially searched in our database all nodules cytologically classified as indeterminate after the systematic introduction of ICCRTC at our institute (December 2014) and the last search was performed on 30 April 2018. Then, we selected for the present study only lesions undergone both FNA and final histology at our institutes. At our institute, thyroid nodules with indeterminate FNA report are generally addressed to surgery in presence of ultrasound or clinical features suspicious for malignancy or when they are within a large goiter with compressive symptoms. The study was approved by Ethical Committee of Ospedale S. Andrea of Rome.

### Gold standard of the study

Histologic diagnosis was adopted as gold standard in all cases.

### Statistical analysis

The prevalence of cancers among the categories Tir 3A and Tir 3B was compared by chi square or Fisher exact test, when indicated. Continuous variables (such as patients' age and nodules' size) were compared by Mann-Whitney non-parametric test. Incidentally discovered cancers (i.e., microcarcinomas found in another nodule) were not considered for statistical analysis. Statistical significance was set at *p* = 0.05. All statistical analyses were performed by MedCalc (MedCalc Software bvba, Belgium).

## Results

According to the selection criteria a number of 157 nodules with indeterminate FNA, of which 58 Tir 3A and 99 Tir 3B was initially found in our registry. After exclusion of those cases not yet resected (*n* = 82), a series of 75 indeterminate lesions from 71 patients (46 female, 25 male, mean age was 52 year) were included as study series. At final histology 25 (33.3%) carcinomas and 50 (67.7%) benign lesions were found. No significant difference (*p* = 0.48) was found in cancer rate between females (20/46, 43.4%) and males (8/25, 32%). The mean age in the cancer group did not significantly differ from that of benign group (mean age 52 yr in both). No significant difference was found in mean nodules' size being 19.6 mm in cancers and 22.7 mm in benign lesions. Table 1 details features of the series.

**Table 1:** Main characteristic of the series.

**Nodule/patients with histologic outcome (*n*)**	**75 nodules/71 patients**
Females/males (*n*)	46/25
Mean age (yr)	52.3
Tir 3B/Tir 3A (*n*)	50/25
Mean size of nodules (mm)	21.2
Cancers/benign lesions at histology (*n*)	25/50

*The final series of 75 lesions with histologic follow-up was collected among a series of 157 cases with indeterminate FNA recorded during the study period*.

**Figure 1:**
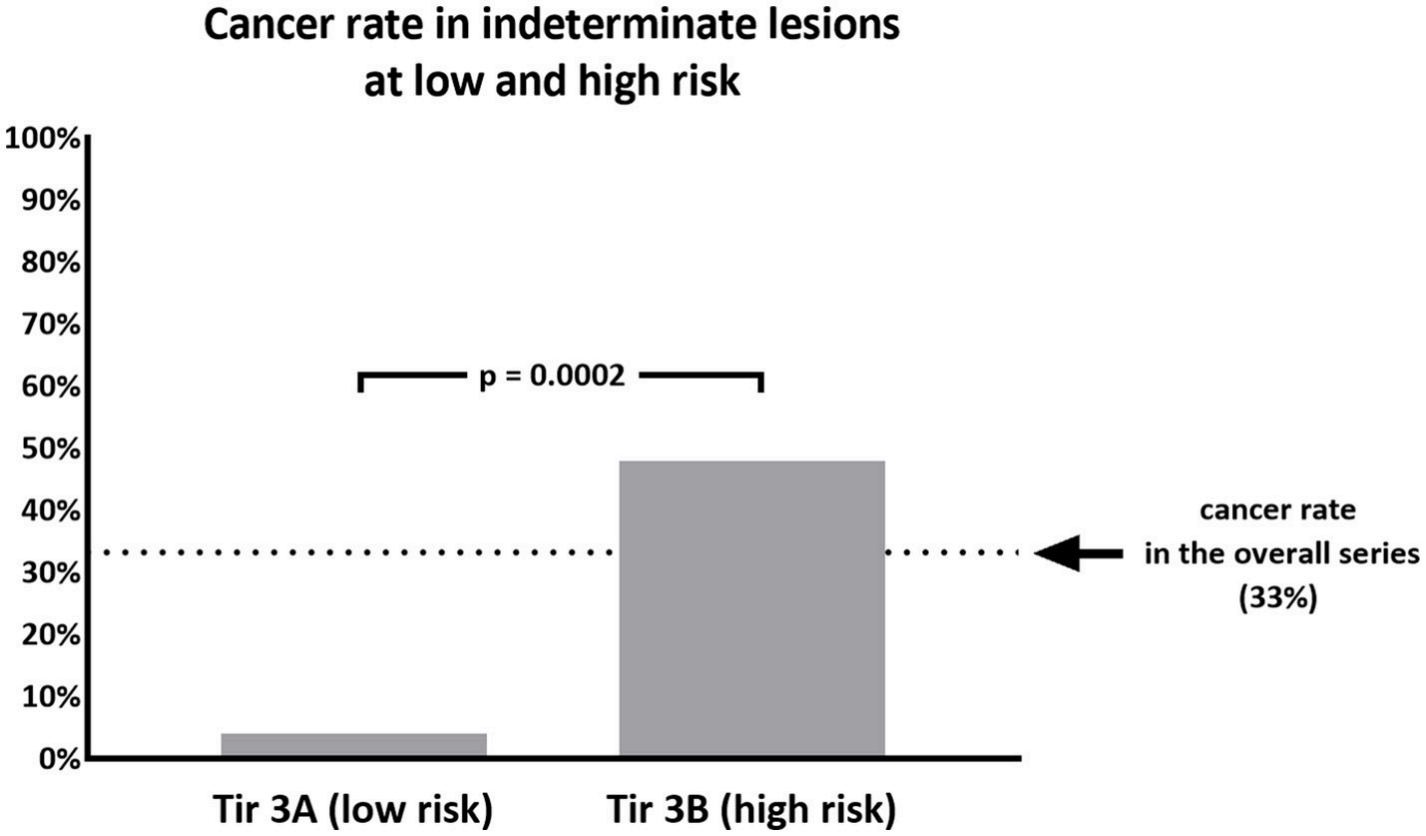
Percentage of malignant nodules recorded in 75 thyroid nodules cytologically classified as Tir 3A (low risk) or Tir 3B (high risk).

When we analyzed the subcategories of low and high risk, we found 25 nodules classified as Tir 3A and 50 as Tir 3B. As a significant difference, the cancer rate observed in Tir 3A (1/25, 4%) was significantly (*p* = 0.0002) lower than that of Tir 3B (24/50, 48%) (Figure 1). No significant differences were found in the age and size between the two subcategories. Among the malignant lesions, 21 papillary carcinomas (all in Tir 3B group), 2 follicular carcinomas (1 in Tir 3B and 1 in Tir 3A), and 2 medullary carcinomas (all in Tir 3B). In the latter cases calcitonin value was not available at the moment of cytologic examination. Table 2 reports details of histologic type/subtype of cancers and ATA risk assessment of differentiated ones. Between the benign lesions there were 10 follicular adenomas (4 in Tir 3B and 6 in Tir 3A), 9 oncocytic adenomas (7 in Tir 3B and 2 in Tir 3A), 20 adenomatous hyperplasias (11 in Tir 3B and 9 in Tir 3A), and 9 thyroiditis hyperplasias (4 in Tir 3B and 5 in Tir 3A).

**Table 2:** Histologic type and ATA risk assessment of differentiated carcinomas of the series.

	**Tir 3A**	**Tir 3B**
**Histologic type of carcinomas (*****n*****)**		
Follicular variant of papillary carcinoma	–	12
Classic variant of papillary carcinoma	–	6
Oncocytic variant of papillary carcinoma	–	2
Poorly differentiated carcinoma	–	1
Follicular carcinoma	1	1
Medullary carcinoma	–	2
**ATA risk class of differentiated carcinomas (*****n*****)**		
Low risk	1	14
Intermediate risk	–	8
High risk	–	2

*The final series of cancer included 23 differentiated carcinomas and 2 medullary carcinomas. Risk assessment was made according to ATA guidelines (2)*.

## Discussion

**Figure 2:**
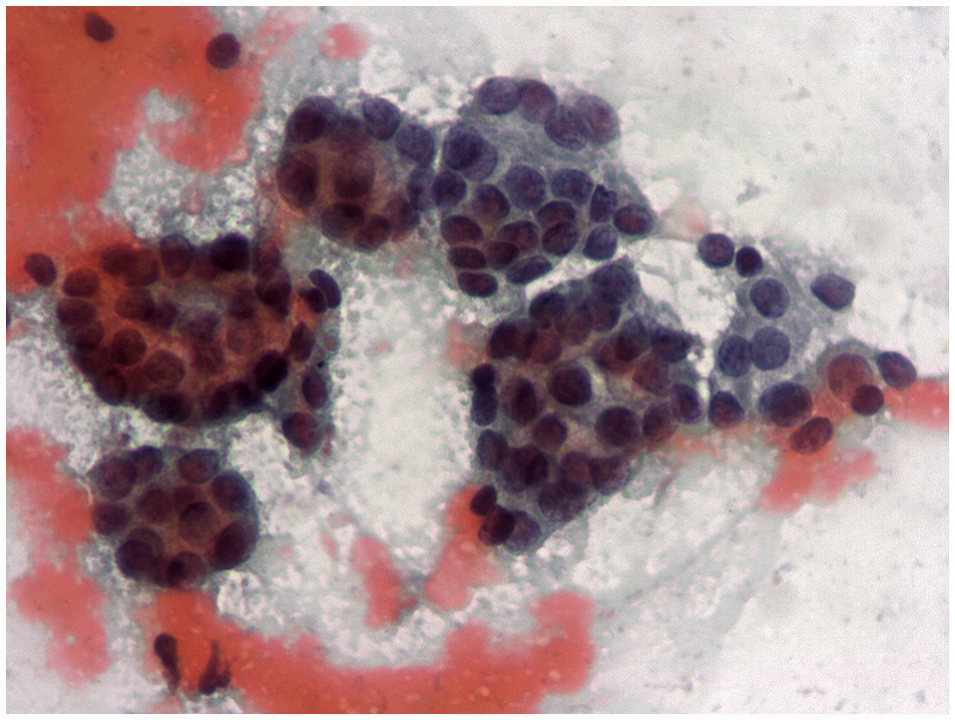
One centimeter solid hypoechoic thyroid nodule in a 65 year old woman cytologically classified as Tir 3A. Follicular proliferation on a bloody background (400X).

**Figure 3:**
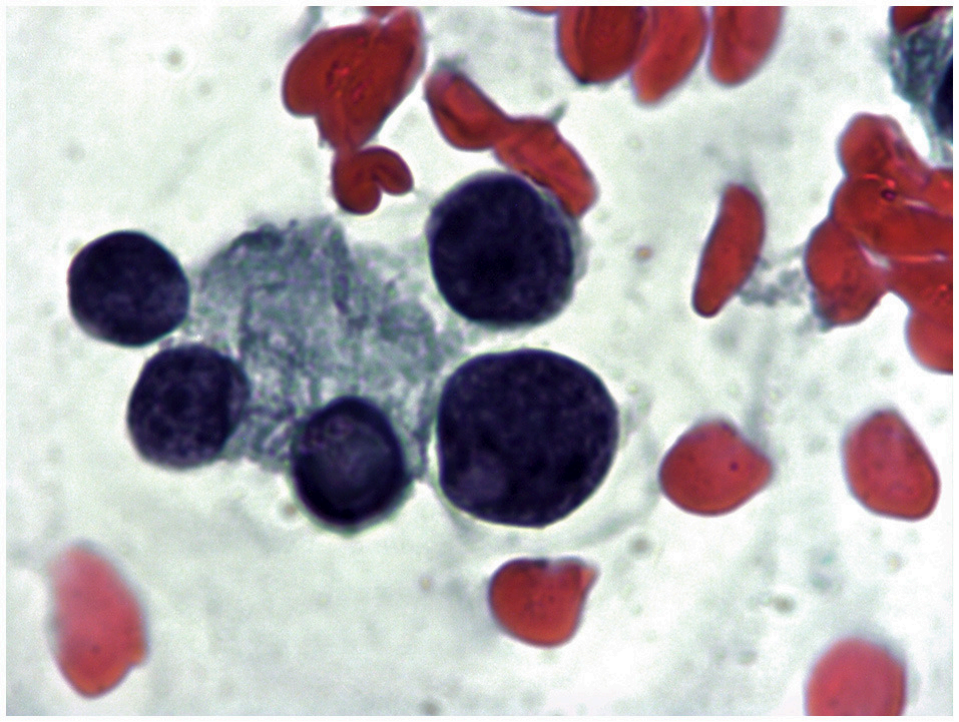
Mixed hypoechoic thyroid nodule 1.5 cm in major diameter in a 72 year old woman cytologically classified as Tir 3B. A follicle with focal nuclear clearing on a mixed colloid and hematic background (1000X).

The major challenge of current international guidelines for thyroid cytology (15–17) is the discrimination of nodules with indeterminate pattern at low and high risk of malignancy. This issue is crucial to address patients to surgical resection when they have a high risk (Tir 3B, Thy 3f, Category IV) or to clinical and ultrasonographic follow-up in presence of low risk (Tir 3A, Thy 3a, Category III). The most recent literature has showed that ICCRTC (17) can play a significant role in managing patients with indeterminate FNA report due to optimal performance to assess these lesions at low and high risk (18). The results of that meta-analysis (18) were obtained by pooling data from studies including nodules re-classified retrospectively according to ICCRTC (19–24). However, similar results were recorded later in papers including nodules prospectively classified according to ICCRTC (25, 26). Our present data, recorded in a series of indeterminate nodules entirely classified by ICCRTC during the clinical practice, corroborate that previously observed by the other Italian and Switzerland institutes (19–26). Figures 2, 3 illustrate the cytologic presentation of two cases of the series. In fact, a significant difference of cancer rate was observed between Tir 3A and Tir 3B. As a strength of the present findings, the rate of malignancy of the overall series (33.3%) was in line with that one would expect in a group of indeterminate thyroid nodules undergone surgery (Figure 1). On the other hand, the different cancer rate reported in different institutes (19–26) reflects the specific institutional approach to indeterminate lesions; low cancer rate should depend on a high percentage of patients submitted to surgery. Following the present data and the above published papers (19–26), ICCRTC has to be considered as reliable to manage indeterminate lesions in the clinical practice. In this context, no high reliability of the Bethesda system (16) was recorded in a meta-analysis, and significant difference was reported by Rullo et al. (27) in a large series of indeterminate nodules retrospectively re-classified according to both ICCRTC (17) and Bethesda system (16); there, the rate of malignancy in the AUS-FLUS (category III) was significantly higher than in Tir 3A, while there were no differences between FN/SFN (category IV) and Tir 3B. In addition, suboptimal performance of Bethesda system was recorded in a previous meta-analysis combining large series of studies with nodules classified as Category III or IV (28). These limits of the Bethesda system should be solved by its revised version (29). This issue indirectly supports the rationale of ICCRTC 2014 (17) and advices for new papers and new meta-analyses to further evaluate the cancer risk of the categories III and IV. All in all, the excellent performance of ICCRTC in identifying low and high risk indeterminate lesions could reach a significant interest for clinical practice, especially in patients with low risk who may be followed-up over time (30).

Some potential limits of the present study should be discussed. Present data report a series of 75 nodules undergone final diagnostic surgery among a large series of 157 cases with indeterminate FNA report; this study design could introduce a selection bias and then reduce the statistical strength of results. On the other hand, all cases included in the final series had been prospectively classified as Tir 3A or Tir 3B by pathologists with large experience of more than 20 years and managed by clinicians consequently. Certainly, other clinical features might have influenced the decisional planning of surgical treatment. A prospective multicenter trial remains the optimal study design in this context.

In conclusion, the present study confirms that the ICCRTC guidelines discriminate efficiently indeterminate lesions at low risk of malignancy from those at high risk and could be used confidentially in clinical practice.

## Ethics statement

This study was carried out in accordance with the recommendations of Ethical Committee of S. Andrea Hospital of Rome. The protocol was approved by this committee. All subjects gave written informed consent in accordance with the Declaration of Helsinki.

## Author contributions

PT and SV conceived and designed the study. GS collected data. MG made the cytological diagnosis. SV, MG, FD, CB, PA, LI and GS took care of the casuistry. PT and FR analyzed the data. PT and SV wrote the paper. All authors contributed to revise critically data and full manuscript.

### Conflict of interest statement

The authors declare that the research was conducted in the absence of any commercial or financial relationships that could be construed as a potential conflict of interest.

## References

[1] Gharib H Papini E Paschke R Duick DS Valcavi R Hegedüs L. American Association of Clinical Endocrinologists, Associazione Medici Endocrinologi, and European Thyroid Association Medical Guidelines for clinical practice for the diagnosis and management of thyroid nodules. Endocr Pract. (2010) 16:1–43. 10.4158/10024.GL 20497938

[2] Haugen BR Alexander EK Bible KC Doherty GM Mandel SJ Nikiforov YE. 2015 American Thyroid Association Management Guidelines for Adult Patients with Thyroid Nodules and Differentiated Thyroid Cancer: the American Thyroid Association Guidelines Task Force on Thyroid Nodules and Differentiated Thyroid Cancer. Thyroid (2016) 26:1–133. 10.1089/thy.2015.0020 26462967 PMC4739132

[3] Baloch ZW Fleisher S LiVolsi VA Gupta PK. Diagnosis of “follicular neoplasm”: a gray zone in thyroid fine-needle aspiration cytology. Diagn Cytopathol. (2002) 26:41–4. 10.1002/dc.10043 11782086

[4] Trimboli P Treglia G Guidobaldi L Saggiorato E Nigri G Crescenzi A. Clinical characteristics as predictors of malignancy in patients with indeterminate thyroid cytology: a meta-analysis. Endocrine (2014) 46:52–9. 10.1007/s12020-013-0057-1 24197803

[5] Sciacchitano S Lavra L Ulivieri A Magi F De Francesco GP Bellotti C. Comparative analysis of diagnostic performance, feasibility and cost of different test-methods for thyroid nodules with indeterminate cytology. Oncotarget (2017) 8:49421–42. 10.18632/oncotarget.17220 28472764 PMC5564779

[6] Heinzel A Müller D Behrendt FF Giovanella L Mottaghy FM Verburg FA. Thyroid nodules with indeterminate cytology: molecular imaging with ^99m^Tc-methoxyisobutylisonitrile (MIBI) is more cost-effective than the Afirma gene expression classifier. Eur J Nucl Med Mol Imaging (2014) 41:1497–500. 10.1007/s00259-014-2760-4 24705621

[7] Piccardo A Puntoni M Treglia G Foppiani L Bertagna F Paparo F. Thyroid nodules with indeterminate cytology: prospective comparison between 18F-FDG-PET/CT, multiparametric neck ultrasonography, 99mTc-MIBI scintigraphy and histology. Eur J Endocrinol. (2016) 174:693–703. 10.1530/EJE-15-1199 26966173

[8] Bartolazzi A Orlandi F Saggiorato E Volante M Arecco F Rossetto R. (2008). Galectin-3-expression analysis in the surgical selection of follicular thyroid nodules with indeterminate fine-needle aspiration cytology: a prospective multicentre study. Lancet Oncol. 9:543–9. 10.1016/S1470-2045(08)70132-3 18495537

[9] Trimboli P Treglia G Condorelli E Romanelli F Crescenzi A Bongiovanni M. BRAF mutated carcinomas among thyroid nodules with prior indeterminate FNA report: a systematic review and meta-analysis. Clin Endocinol. (2016) 84:315–20. 10.1111/cen.12806 25920006

[10] Trimboli P Treglia G Sadeghi R Romanelli F Giovanella L. Reliability of real-time elastography to diagnose thyroid nodules previously read at FNAC as indeterminate: a meta-analysis. Endocrine (2015) 50:335–43. 10.1007/s12020-014-0510-9 25534701

[11] Bartolazzi A Sciacchitano S D'Alessandria C. Galectin-3: the impact on the clinical management of patients with thyroid nodules and future perspectives. Int J Mol Sci. (2018) 19:E445. 10.3390/ijms19020445 29393868 PMC5855667

[12] Trimboli P Fulciniti F Zilioli V Ceriani L Giovanella L. Accuracy of international ultrasound risk stratification systems in thyroid lesions cytologically classified as indeterminate. Diagn Cytopathol. (2017) 45:113–7. 10.1002/dc.23651 28024119

[13] Trimboli P Deandrea M Mormile A Ceriani L Garino F Limone PP. American Thyroid Association ultrasound system for the initial assessment of thyroid nodules: use in stratifying the risk of malignancy of indeterminate lesions. Head Neck (2018) 40:722–7. 10.1002/hed.25038 29247582

[14] Alexander EK Kennedy GC Baloch ZW Cibas ES Chudova D Diggans J. Preoperative diagnosis of benign thyroid nodules with indeterminate cytology. N Engl J Med. (2012) 367:705–15. 10.1056/NEJMoa1203208 22731672

[15] Perros P Boelaert K Colley S Evans C Evans RM Gerrard Ba G British Thyroid Association. Guidelines for the management of thyroid cancer. Clin Endocrinol. (2014) 81:1–122. 10.1111/cen.12515 24989897

[16] Cibas ES Ali SZ. The Bethesda System for reporting thyroid cytopathology. Thyroid (2009) 19:1159–65. 10.1089/thy.2009.0274 19888858

[17] Nardi F Basolo F Crescenzi A Fadda G Frasoldati A Orlandi F. Italian consensus for the classification and reporting of thyroid cytology. J Endocrinol Invest. (2014) 37:593–9. 10.1007/s40618-014-0062-0 24789536

[18] Trimboli P Crescenzi A Giovanella L. Performance of Italian Consensus for the Classification and Reporting of Thyroid Cytology (ICCRTC) in discriminating indeterminate lesions at low and high risk of malignancy. A systematic review and meta-analysis. Endocrine (2018) 60:31–5. 10.1007/s12020-017-1382-6 28786076

[19] Trimboli P Guidobaldi L Amendola S Nasrollah N Romanelli F Attanasio D. Galectin-3 and HBME-1 improve the accuracy of core biopsy in indeterminate thyroid nodules. Endocrine (2016) 52:39–45. 10.1007/s12020-015-0678-7 26142180

[20] Tartaglia F Giuliani A Tromba L Carbotta S Karpathiotakis M Tortorelli G. Fine needle aspiration cytology of 650 thyroid nodules operated for multinodular goiter: a cyto-histological correlation based on the new Italian cytological classification (siapec 2014). J Biol Regul Homeost Agents (2016) 30:1187–93. 28078873

[21] Grani G Lamartina L Ascoli V Bosco D Nardi F D'Ambrosio F. Ultrasonography scoring systems can rule out malignancy in cytologically indeterminate thyroid nodules. Endocrine (2017) 57:256–61. 10.1007/s12020-016-1148-6 27804016

[22] Quaglino F Marchese V Mazza E Gottero C Lemini R Taraglio S. When is thyroidectomy the right choice? comparison between fine-needle aspiration and final histology in a single institution experience. Eur Thyroid J. (2017) 6:94–100. 10.1159/000452622 28589091 PMC5422848

[23] Ulisse S Bosco D Nardi F Nesca A D'Armiento E Guglielmino V. Thyroid imaging reporting and data system score combined with the new Italian classification for thyroid cytology improves the clinical management of indeterminate nodules. Int J Endocrinol. (2017) 2017:9692304. 10.1155/2017/9692304 28348589 PMC5350532

[24] Medas F Erdas E Gordini L Conzo G Gambardella C Canu GL. Risk of malignancy in thyroid nodules classified as TIR-3A: what therapy? Int J Surg. (2017) 41:S60–4. 10.1016/j.ijsu.2017.03.056 28506415

[25] Trimboli P Fulciniti F Merlo E Barizzi J Mazzucchelli L Giovanella L. Histologic outcome of indeterminate thyroid nodules classified at low or high risk. Endocr Pathol. (2018) 29:75–9. 10.1007/s12022-018-9517-8 29396808

[26] Lauria A Maddaloni E Briganti SI Beretta Anguissola G Perrella E Taffon C Differences between ATA, AACE/ACE/AME and ACR TI-RADS ultrasound classifications performance in identifying cytological high-risk thyroid nodules. Eur J Endocrinol. (2018) 78:595–603. 10.1530/EJE-18-0083 29626008

[27] Rullo E Minelli G Bosco D Nardi F Ascoli V. Evaluation of the Italian cytological subclassification of thyroid indeterminate nodules into TIR-3A and TIR-3B: a retrospective study of 290 cases with histological correlation from a single institution. J Endocrinol Invest. (2018) 41:531–8. 10.1007/s40618-017-0763-2 28948534

[28] Straccia P Rossi ED Bizzarro T Brunelli C Cianfrini F Damiani D Fadda G. A meta-analytic review of the Bethesda System for Reporting Thyroid Cytopathology: has the rate of malignancy in indeterminate lesions been underestimated? Cancer Cytopathol. (2015) 123:713–22. 10.1002/cncy.21605 26355876

[29] Ali SZ Baloch ES The Bethesda System for Reporting Thyroid Cytopathology. Cham: Springer International Publishing AG (2018).

[30] Sciacchitano S Lavra L Ulivieri A Magi F Porcelli T Amendola S. Combined clinical and ultrasound follow-up assists in malignancy detection in Galectin-3 negative Thy-3 thyroid nodules. Endocrine (2016) 54:139–47. 10.1007/s12020-015-0774-8 26475496

